# Polydopamine-assisted lithium ion loading on titanium alloy synergistically enhances osteogenesis and immunomodulation for bone regeneration: an *In Vitro* study

**DOI:** 10.1186/s12903-026-08184-y

**Published:** 2026-03-31

**Authors:** Quan Zhong, Zihe Yu, Zechuan Zhou, Lan Luo, Chao Chen, Kai Luo, Chongwu Liu

**Affiliations:** 1 Clinical Research Center for Oral Tissue Deficiency Diseases of Fujian Province & Fujian Key Laboratory of Oral Diseases & Fujian Provincial Engineering Research Center of Oral Biomaterial, School and Hospital of Stomatology, Fujian Medical University, Fuzhou, China; 2 Stomatological Key Laboratory of Fujian College and University & Institute of Stomatology & Center of Oral Tissue Engineering, School and Hospital of Stomatology, Fujian Medical University, Fuzhou, China; 3 Fuzhou First General Hospital, Fuzhou, China

**Keywords:** Polydopamine, Lithium ion, Titanium alloy, Bone regeneration, Osteoimmunology

## Abstract

**Background:**

​Periodontal bone defects present significant challenges in clinical regeneration. Titanium mesh has been widely used in guided bone regeneration (GBR) due to its excellent mechanical properties, but its bioinert nature and potential immune response limit its regenerative efficacy. Surface modification of titanium alloys represents a promising approach to enhance their bioactivity. This study explores a novel strategy using polydopamine (PDA)-mediated lithium (Li) ion (Li^+^)loading on titanium surfaces to simultaneously promote osteogenesis and immunomodulation.

**Methods:**

​Titanium discs were modified through alkali-thermal treatment, PDA coating, and lithium chloride(LiCl) immersion at different concentrations (1, 5, and 10 mol/L). Surface characterization was performed using scanning electron microscopy (SEM) and X-ray photoelectron spectroscopy (XPS). Li^+^ release kinetics and surface hydrophilicity were systematically evaluated. In vitro biological effects were assessed using rat bone marrow stromal cells (rBMSCs) and RAW264.7 macrophages through comprehensive analyses of cell adhesion, proliferation, osteogenic differentiation (Alkaline phosphatase (ALP) activity, mineralization, and gene expression), and macrophage polarization (inflammatory cytokine profiling). Conditioned medium(CM) collected from the co‑culture of macrophages and Ti‑PDA‑5Li was further used to evaluate the effects of the immune microenvironment on the proliferation and osteogenic activity of bone marrow stromal cells (BMSCs).

**Results:**

​The modified surfaces exhibited a well-defined porous nanostructure, with Li^+^ incorporation significantly enhancing surface bioactivity. The Ti-PDA-5Li group demonstrated optimal performance with sustained Li^+^ release and improved hydrophilicity. This group significantly promoted rBMSC adhesion, proliferation, and osteogenic differentiation, showing enhanced ALP activity, mineralized nodule formation, and upregulation of osteogenic markers (*Runx2*,* OCN*,* BSP*,* OPN*). Moreover, Ti-PDA-5Li effectively modulated macrophage polarization towards the anti-inflammatory M2 phenotype, characterized by suppressed the level of pro-inflammatory cytokines (IL-6, TNF-α) and enhanced the level of anti-inflammatory factors (IL-10). CM from macrophages cultured on Ti-PDA-5Li further stimulated osteogenic differentiation in rBMSCs, confirming the immunomodulatory-osteogenic coupling effect.

**Conclusions:**

​The PDA-assisted Li^+^ loading strategy successfully creates a bioactive titanium coating that synergistically promotes bone regeneration through direct osteogenic stimulation and immune microenvironment regulation. This study provides new insights into the development of multifunctional titanium implants for bone regeneration, highlighting the importance of combined osteo-immunomodulatory approaches.

## Introduction

Chronic periodontitis represents a prevalent inflammatory disease characterized by the progressive destruction of periodontal supporting tissues, including the gingiva, alveolar bone, periodontal ligament, and cementum [1]. This pathological process ultimately leads to tooth mobility and loss, significantly impairing patients’ masticatory function, aesthetics, and overall quality of life. The primary objectives of periodontal therapy encompass infection control and the functional and aesthetic restoration of compromised or edentulous areas. However, advanced periodontitis often results in substantial soft and hard tissue deficiencies, presenting considerable challenges for achieving predictable physiological reconstruction [2, 3]. Among these challenges, the regeneration of hard tissues particularly in cases of severe horizontal and vertical bone defects is a critical prerequisite for successful periodontal rehabilitation.

Titanium mesh has gained widespread application in the reconstruction of such bone defects owing to its exceptional mechanical properties and reliable space-maintaining capacity [4]. Its superior malleability facilitates adaptation to complex anatomical morphologies, establishing it as an indispensable component in GBR protocols [5]. Although titanium alloys demonstrate favorable biocompatibility, mechanical strength, and corrosion resistance, several limitations persist in extensive clinical use. For instance, the inherent biological inertness of the titanium oxide layer may compromise peri-implant osteogenesis. Furthermore, the transient immune-inflammatory response elicited by titanium mesh implantation can lead to undesirable bone resorption or degradation of adjacent bone graft materials [6, 7].

Surface modification of titanium alloys represents a promising strategy to address these critical issues [8]. Current modification approaches primarily involve the application of biocompatible coatings or direct alterations to the material’s surface chemistry and topography. The efficacy of such modifications hinges on two key factors: (1) the selection of bioactive components that dictate biological performance, and (2) the interfacial bonding methodology that ensures coating stability and sustained bioactivity [9].

In recent years, PDA has emerged as a versatile platform for surface functionalization and bioactive molecule delivery. The discovery of PDA originated from investigations into mussel adhesion mechanisms. In the 1980s, Waite et al. identified that mussel foot proteins contain high concentrations of 3,4-dihydroxyphenylalanine (DOPA), whose catechol groups are instrumental in surface adhesion [10]. Dopamine (3,4-dihydroxyphenethylamine, DA), a key derivative of DOPA, undergoes oxidative self-polymerization under alkaline conditions to form PDA, which exhibits robust adhesion to various material surfaces including metals, ceramics, and polymers [11, 12]. This adhesive property enables the functionalization of titanium alloys with bioactive ions such as copper and manganese to impart antibacterial properties [13], or with cytokines like IL-4 to enhance osseointegration through immunomodulation [14].

Li, the lightest metallic element, exhibits significant biological activity through its ions beyond their well-established role in treating bipolar disorder [15]. Mechanistically, Li^+^ functions as a potent inhibitor of glycogen synthase kinase-3β (GSK3β), thereby activating the canonical Wnt/β-catenin signaling pathway. This activation promotes the proliferation and osteogenic differentiation of BMSCs and osteoblasts [16], while simultaneously suppressing osteoclastogenesis [17]. Additionally, Li^+^ exhibits immunomodulatory properties that can mitigate inflammation-induced tissue damage [18]. These dual osteogenic and anti-inflammatory characteristics position Li as an attractive candidate for titanium surface modification. Current methodologies for Li^+^ incorporation on titanium surfaces predominantly rely on electrochemical deposition and microarc oxidation techniques [19, 20]. However, electrochemical deposition requires stringent metal pretreatment, while microarc oxidation is highly sensitive to equipment and process parameters [9]. To address these shortcomings, this study will propose a PDA-assisted loading strategy, which remains unexplored as a carrier system for Li⁺ delivery on titanium.

**Fig. 1:**
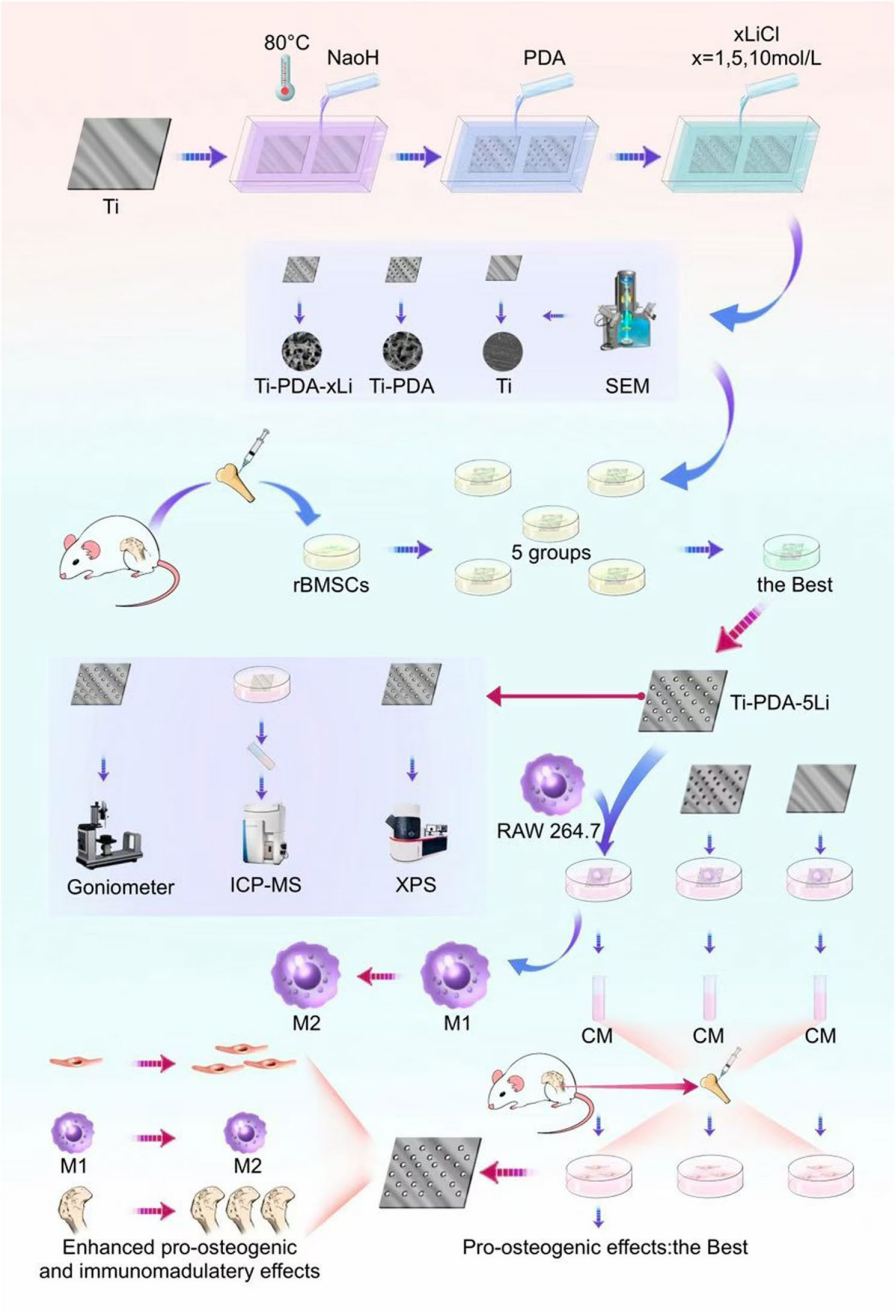
Schematic illustration of PDA-mediated Li^+^ loading on a titanium surface for synergistic enhancement of osteogenesis and immunomodulation. Alkali-heat treated titanium (Ti-6Al-4V) disks were functionalized with a PDA coating to facilitate the subsequent loading of Li^+^ at various concentrations. Surface morphology was analyzed by SEM. Following co-culture with rBMSCs, the sample loaded with 5 mol/L LiCl (denoted as Ti-PDA-5Li) was identified as optimal and was further characterized for its surface hydrophilicity and Li^+^ release profile. When co-cultured with RAW264.7 macrophages, the Ti-PDA-5Li substrate promoted a shift in macrophage polarization from the pro-inflammatory M1 phenotype toward the anti-inflammatory M2 phenotype. CM collected from macrophages cultured on Ti-PDA-5Li was then used to treat rBMSCs, which significantly enhanced their osteogenic differentiation compared to conditioned media from control groups

Building upon our previous research foundation [21–23], this study aims to develop a novel Li^+^-functionalized titanium coating via PDA-mediated surface modification. We seek to identify the optimal Li^+^ concentration for enhanced bioactivity, systematically evaluate the osteogenic, anti-inflammatory, and immunomodulatory properties of the Li-PDA coating system(Fig. 1), and provide experimental evidence to support its potential application in GBR procedures for reconstructing severe periodontitis-related bone defects.

## Materials and methods

### Preparation and surface characterization of Li^+^ loaded titanium alloy

Commercially titanium discs (Ti-6Al-4V, 10 × 10 × 2 mm) were sequentially polished with silicon carbide sandpaper ranging from 400 to 5000 grit. The polished samples underwent ultrasonic cleaning in acetone, absolute ethanol, 75% ethanol, and deionized water (DI water) to remove surface contaminants, followed by drying at room temperature. These samples were designated as Group Ti.

For alkali-thermal treatment, Group Ti samples were immersed in 5 mol/L NaOH aqueous solution maintained at 80 °C for 12 h. Subsequently, samples were ultrasonicated in DI water and dried. For PDA coating, samples were placed in 24-well plates containing 1 mL of dopamine hydrochloride solution (2 mg/mL in 10 mmol/L Tris-HCl buffer, pH 8.5) and incubated at room temperature for 24 h in the dark. After thorough washing with DI water and drying, PDA-coated samples were labeled as Group Ti-PDA.

Li^+^ loading was performed by immersing Group Ti-PDA samples in LiCl solutions at concentrations of 1, 5, or 10 mol/L with continuous shaking at 80 rpm for 24 h. Unbound Li ions were removed by ultrasonication, resulting in Group Ti-PDA-xLi (where x = 1, 5, 10). Surface morphology and microstructural characteristics were examined using SEM (JSM-IT200, JEOL, Germany) to compare differences among Ti, Ti-PDA, and Ti-PDA-xLi groups.

### Surface chemistry, Li^+^ release, and hydrophilicity analysis

Surface elemental composition was analyzed by XPS using a monochromatic Al Kα X-ray source. Survey scans were conducted at 150 eV pass energy, while high-resolution scans were performed at 50 eV pass energy, with calibration relative to the C 1s peak at 284.8 eV.

Li^+^ release kinetics were evaluated by immersing Ti-PDA-5Li samples in phosphate-buffered saline (PBS) at a surface area-to-volume ratio of 3 cm^2^/mL maintained at 37 °C. Supernatants were collected at predetermined time points (1, 3, 5, and 7 days) and analyzed for Li^+^ concentration using inductively coupled plasma mass spectrometry (ICP-MS).

Hydrophilicity was assessed through static water contact angle measurements using a goniometer (DataPhysics OCA20, Germany). A 2 µL droplet of DI water was deposited on sample surfaces, and contact angles were measured in triplicate for each sample.

### Cell culture and experimental procedures

#### Isolation and culture of rBMSCs

All animal experiments were approved by the Institutional Animal Care and Use Committee (Approval No. IACUC FJMU 2022 − 0572). A total of 10 male Sprague-Dawley rats (6 weeks old, body weight: 220 ± 20 g) were purchased from Shanghai SLAC Laboratory Animal Co., Ltd. Rats were acclimated to laboratory conditions for 7 days prior to experimentation. Deep surgical anesthesia was induced via intraperitoneal injection of a 3% sodium pentobarbital solution (45 mg/kg), with anesthetic depth confirmed by loss of corneal reflex and absence of withdrawal response to toe pinch. Bilateral femurs were aseptically and promptly harvested from the unconscious animals for subsequent isolation of bone marrow stromal cells [24]. Immediately following tissue collection, euthanasia was performed by an intraperitoneal overdose of sodium pentobarbital (150 mg/kg). Cells were cultured in α-MEM medium supplemented with 15% fetal bovine serum (FBS; Hyclone, USA), 100 U/mL penicillin, and 100 µg/mL streptomycin at 37 °C in a humidified atmosphere containing 5% CO_2_. Cells were identified based on previous studies from the research group and cells at passage 3 were used for all experiments [25].

#### Macrophage culture

RAW264.7 murine macrophage cells were obtained from the Chinese Academy of Sciences Cell Bank and maintained in DMEM supplemented with 10% FBS and 1% penicillin-streptomycin at 37 °C in 5% CO_2_.

### Biological characterization

#### Effects on rBMSCs adhesion and proliferation

Sterilized samples (Ti, Ti-PDA, Ti-PDA-xLi) were placed in 24-well plates, and rBMSCs were seeded at a density of 2 × 10^4^cells/well. Cell adhesion was evaluated through cytoskeleton staining using rhodamine-phalloidin (1:200 dilution) after 24 h of culture, followed by observation under fluorescence microscopy.

Cell viability was assessed using calcein-AM (2 µmol/L, green fluorescence for live cells) and propidium iodide (4 µmol/L, red fluorescence for dead cells) staining. The percentage of live cells was calculated from three independent experiments. Cell proliferation was quantified using Cell Counting Kit-8 (CCK-8; Dojindo, Japan) assay at day 1, 3, and 7 according to the manufacturer’s instructions.

#### Osteogenic differentiation of rBMSCs

rBMSCs were seeded on sample surfaces at 2 × 10^4^cells/well and cultured in osteogenic induction medium containing 10% FBS, 0.1 µmol/L dexamethasone, 50 µmol/L ascorbate-2-phosphate, and 10 mmol/L β-glycerophosphate. ALP activity was evaluated at day 7 using BCIP/NBT staining kit (Beyotime, China) and quantified through enzymatic assay. Mineralization capacity was assessed by Alizarin Red S (ARS) staining at day 14 to detect calcium nodule formation. Osteogenic gene expression, such as, runt-related transcription factor 2 (*Runx2*), osteocalcin (*OCN*), bone sialoprotein *(BSP*), and osteopontin (*OPN*), were analyzed by quantitative real-time PCR (qRT-PCR) at day 7, with glyceraldehyde-3-phosphate dehydrogenase (*GAPDH*) serving as the internal control.

#### Macrophage response to modified surfaces

RAW264.7 cells were seeded at 8 × 10⁴ cells/well on sterilized samples (Ti, Ti-PDA, Ti-PDA-5Li). Cell adhesion and proliferation were evaluated using cytoskeleton staining (day 1 and 3) and CCK-8 assay (day 1, 3, and 5), respectively. Inflammatory marker expression, such as, interleukin-6 (*IL-6*), interleukin-10 (*IL-10*), tumor necrosis factor-α (*TNF-α*), inducible nitric oxide synthase (*iNOS*), arginase-1 (*Arg-1*), were analyzed by qRT-PCR at day 3.

#### CM-induced osteogenesis in rBMSCs

RAW264.7 cells (8 × 10^4^ cells/well) were cultured on sample surfaces for 3 days. CM was collected by centrifugation of culture supernatants. The CM was diluted 1:1 with fresh osteogenic medium to generate Ti-CM, Ti-PDA-CM, and Ti-PDA-5Li-CM. The osteogenic potential of rBMSCs induced by CM was assessed through ALP activity (day 7), ARS staining (day 14), and osteogenic gene expression (*ALP*,* Runx2*, collagen type I (*Col-1*) ) analysis at day 7.

### Quantitative real-time PCR analysis

Total RNA was extracted using TRIzol reagent (Invitrogen, USA) according to the manufacturer’s instructions. cDNA synthesis was performed using PrimeScript RT reagent kit (Takara, Japan). qRT-PCR was carried out using SYBR Premix Ex Taq (Takara, Japan) on a StepOnePlus Real-Time PCR System (Applied Biosystems, USA). The amplification conditions were: 95 °C for 30 s, followed by 40 cycles of 95 °C for 5 s and 60 °C for 30 s. Gene expression levels were calculated using the 2^-ΔΔCt^ method normalized to GAPDH. Primer sequences are listed in Table 1 for osteogenesis genes and Table 2 for inflammation and polarization genes.

**Table 1:** Primer sequences for osteogenesis genes used in qPCR analysis

Gene	Forward primer sequence(5’-3’)	Reverse primer sequence(5’-3’)
*GAPDH*	CGGCAAGTTCAACGGCACAGTCAAGG	ACGACATACTCAGCACCAGCATCACC
*Runx2*	ACCAGCAGCACTCCATATCTCTAC	CTTCCATCAGCGTCAACACCAT
*OCN*	GGACCCTCTCTCTGCTCACTCTG	ACCTTACTGCCCTCCTGCTTGG
*BSP​*	ACAACACTGCGTATGAAACCTATGAC	AGTAATAATCCTGACCCTCGTAGCC
*OPN​*	CCGTGGGAAGGACAGTTATG	GCTCATTGCTCTCATCATTGG
*ALP*	ACAAGGTGGTGGACGGTGAAC	CGTGAAGCAGGTGAGCCATAGG
*Col-1​*	CAACAGACTGGCAACCTCAAGAAG	CACAAGCGTGCTGTAGGTGAATC

### Statistical analysis

All data are presented as mean ± standard deviation (SD) from at least three independent experiments. Statistical analysis was performed using SPSS 21.0 software (IBM, USA). Normality and homogeneity of variance were verified using Shapiro-Wilk test and Levene’s test, respectively. Comparisons among multiple groups were conducted using one-way analysis of variance (ANOVA) followed by Tukey’s post hoc test. Statistical significance was defined as *p* < 0.05.

**Table 2:** Primer sequences for inflammation & polarization genes used in qPCR analysis

Gene	Forward primer sequence(5’-3’)	Reverse primer sequence(5’-3’)
*GAPDH*	AGGTGGTGAAGCAGGCATC	AAGGTGGAAGAGTGGGAGTTG
*IL-6*	GAAACCGCTATGAAGTTCCTCTCT	GTATCCTCTGTGAAGTCTCCTCTCC
*IL-10*	CCCCAGCCGCTTCATCCC	ACAAACAATACACCATTCCCAGAGG
*TNF-α*	AAGGGAGAGTGGTCAGGTTGC	TGGAAAGGTCTGAAGGTAGGAAGG
*iNOS*	GCACCACCCTCCTCGTTCAG	CCACAACTCGCTCCAAGATTCC
*Arg-1*	GCCTTTGTTGATGTCCCTAATG	GCACCACACTGACTCTTCC

## Results

### Surface morphology of Li^+^ loaded titanium alloy

**Fig. 2:**
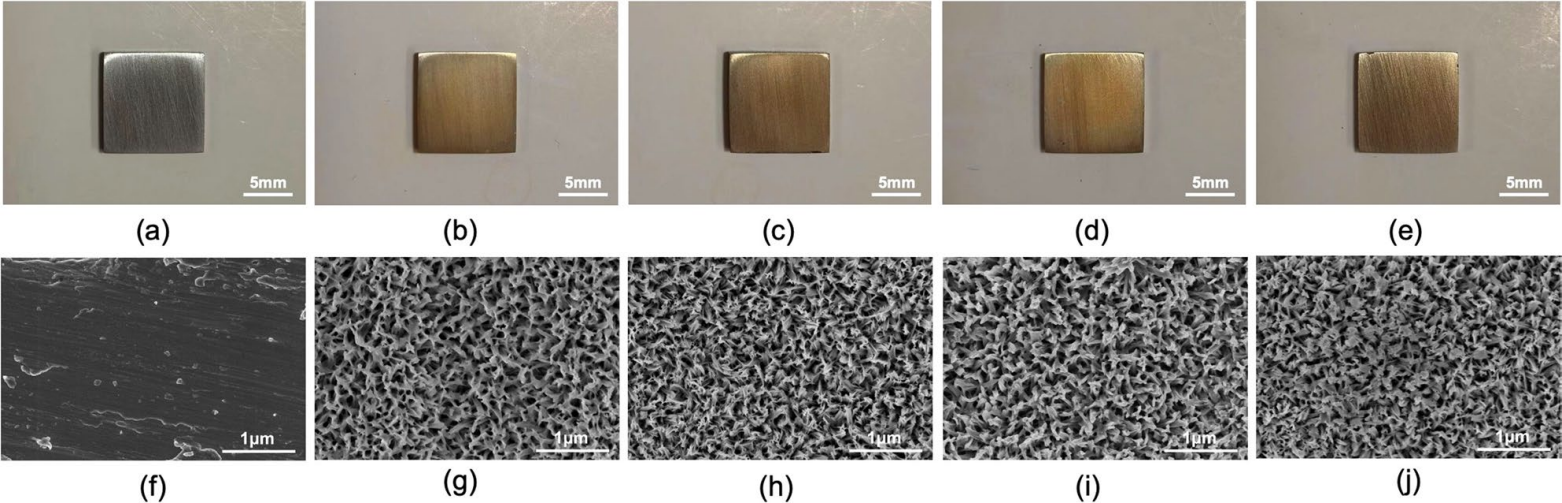
Surface characterization of modified titanium alloys. **a** Pristine Ti surface (gray), **b** Ti-PDA surface (golden), **c-e** Ti-PDA surfaces after loading with 1, 5, and 10 mol/L LiCl, respectively, showing no significant color change, **f** Untreated Ti (smooth, with polishing scratches, SEM), **g** Ti-PDA (3D porous network with ~ 80 nm pores), **h-j** Ti-PDA-xLi surfaces: increasing LiCl concentrations (1–10 mol/L) progressively thickened pore walls and reduced pore diameters while maintaining the porous architecture. (scale bar: a-e: 5 mm; f-j: 1 μm)

The pristine titanium alloy surface exhibited a gray color(Fig. 2a), which turned golden after being coated with PDA(Fig. 2b). Following the loading of different concentrations of LiCl, no discernible colorimetric change was observed on the surface(Panels c-e in Fig. 2 correspond to LiCl concentrations of 1, 5, and 10 mol/L, respectively). SEM revealed distinct surface characteristics among the modified titanium alloys (Fig. 2f–j). The untreated Ti group exhibited a relatively smooth surface with regular unidirectional polishing scratches (Fig. 2f). In contrast, the PDA-coated Ti (Ti-PDA) group displayed a three-dimensional porous network structure with columnar pore walls (approximately 80 nm in diameter) following alkali-thermal treatment and PDA modification (Fig. 2g). This network structure adhered excellently to the substrate without visible fractures or delamination. All Li^+^ loaded groups (Ti-PDA-xLi) maintained an overall porous architecture. Increasing LiCl concentrations resulted in thickened pore walls and reduced pore diameters in a concentration-dependent manner (Fig. 2h–j).

### Li^+^ loaded titanium alloy regulated adhesion and proliferation of rBMSCs

**Fig. 3:**
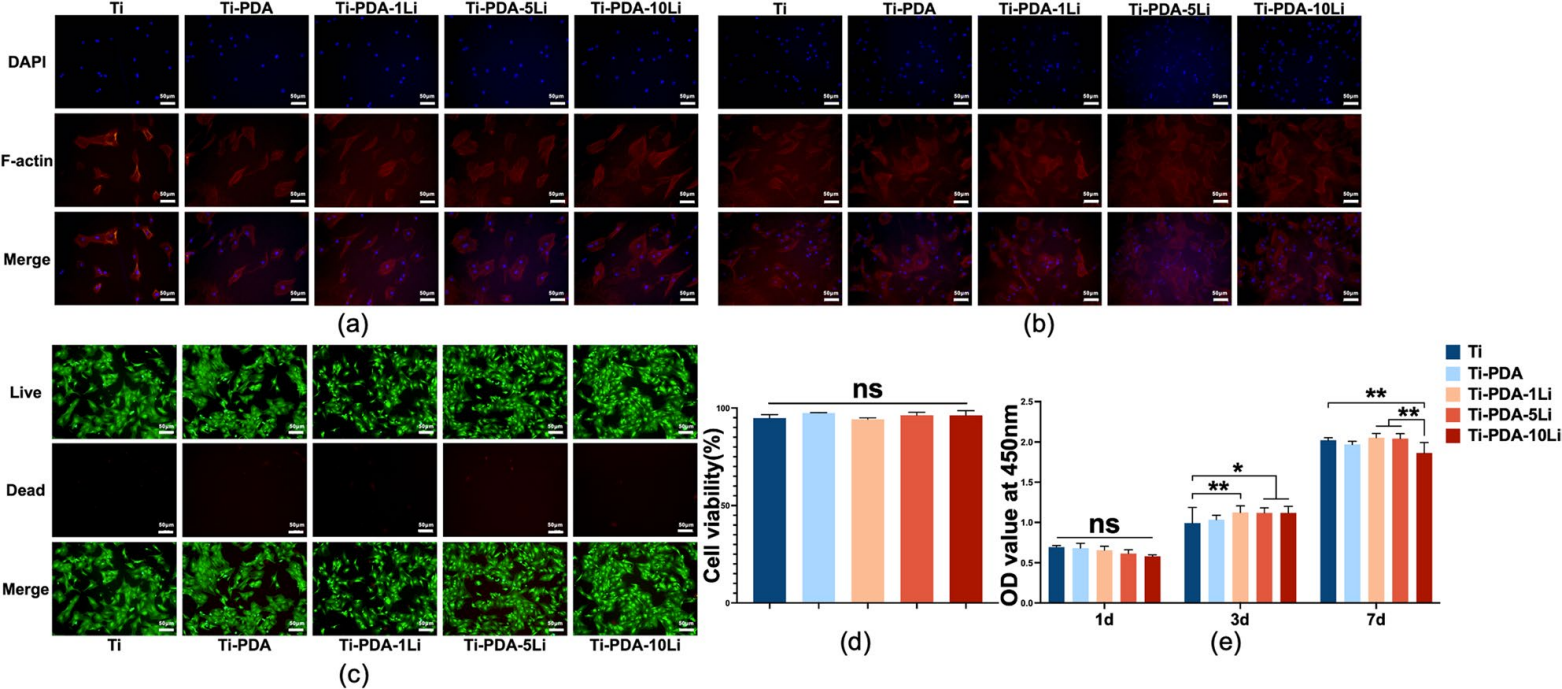
Effects of Li^+^ loaded titanium alloys with varying concentrations on rBMSCs adhesion and proliferation. **a** Cytoskeleton staining after 1-day culture (scale bar: 50 μm), **b** Cytoskeleton staining after 3-day culture (scale bar: 50 μm), **c** Live/dead cell staining (calcein-AM/propidium iodide; scale bar: 50 μm), **d** Quantitative analysis of cell viability, **e** Proliferation rates at day 1, 3, and 7. Data: mean ± SD; ns: no significant, * *p< 0.05*, **: *p* < 0.01(one-way ANOVA)

Significant variations in adhesion and proliferation behavior of rBMSCs were observed among the experimental groups (Fig. 3a–e). On day 1, both Ti-PDA and Ti-PDA-Li surfaces promoted better cell spreading and more filopodia formation compared to the pure Ti group (Fig. 3a). Li^+^ concentration did not significantly affect cell morphology at this early stage. Over time, cells on Ti-PDA and Ti-PDA-Li surfaces exhibited enhanced spreading and adhesion (Fig. 3b). Live/dead staining confirmed excellent biocompatibility, with cell viability exceeding 90% in all groups (Fig. 3c, d). On day 3, all Li^+^ loaded groups supported significantly higher cell numbers than pure Ti (*p* < 0.05). By day 7, the Ti-PDA-10Li group showed reduced proliferation compared to pure Ti (*p* < 0.01), whereas the Ti-PDA-5Li group maintained optimal cell growth (Fig. 3e).

### Li^+^ loaded titanium alloy promoted osteogenic differentiation of rBMSCs

**Fig. 4:**
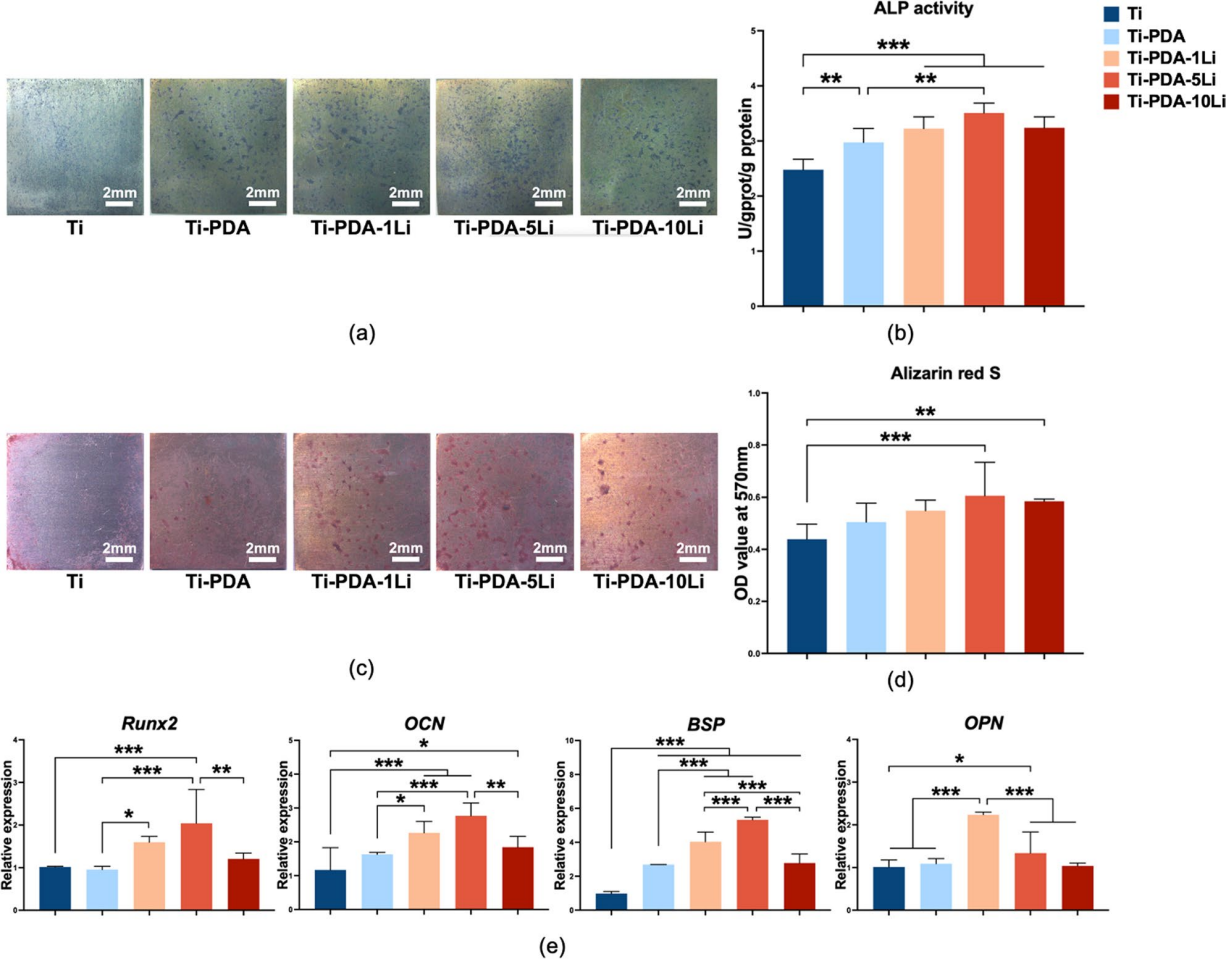
Effects of Li^+^ loaded titanium alloys with varying concentrations on rBMSCs biological activity. **a** ALP staining, **b** Quantitative ALP activity, **c** ARS staining, **d** Quantitative mineralization, **e** Osteogenic gene expression (*Runx2*, *OCN*, *BSP*, *OPN*), (scale bar: 2 mm). Data: mean ± SD; * *p< 0.05*, **: *p* < 0.01, ***: *p* < 0.001 (one-way ANOVA with Tukey’s post-hoc test)

Osteogenic differentiation of rBMSCs was evaluated under various surface conditions. ALP staining revealed intense blue-purple nodules on Ti-PDA and Ti-PDA-Li surfaces (Fig. 4a). Quantitative analysis indicated that the Ti-PDA-5Li group exhibited the highest ALP activity (*p* < 0.01 vs. other groups; Fig. 4b). ARS staining on day 14 demonstrated greater mineralized nodule formation in Ti-PDA-xLi groups (Fig. 4c), with the Ti-PDA-5Li group showing the most pronounced mineralization (Fig. 4d). At the molecular level, Li^+^ loaded groups upregulated the expression of osteogenic markers, such as *Runx*2, *OCN*, *BSP*, and *OPN* on day 7 (Fig. 4e). Among these, Ti-PDA-5Li exhibited the highest expression levels of *Runx2*, *OCN*, and *BSP*.

Based on these results, Li^+^ loaded titanium alloy demonstrated good biocompatibility. Within an appropriate concentration range, it promoted rBMSCs proliferation, enhanced ALP activity and in vitro mineralization capacity, and upregulated osteogenic gene expression. The Ti-PDA-5Li group exhibited the most favorable osteogenic properties and was therefore selected as the sole positive control in subsequent experiments to streamline the research process.

### Surface chemistry, Li^+^ release, and hydrophilicity

**Fig. 5:**
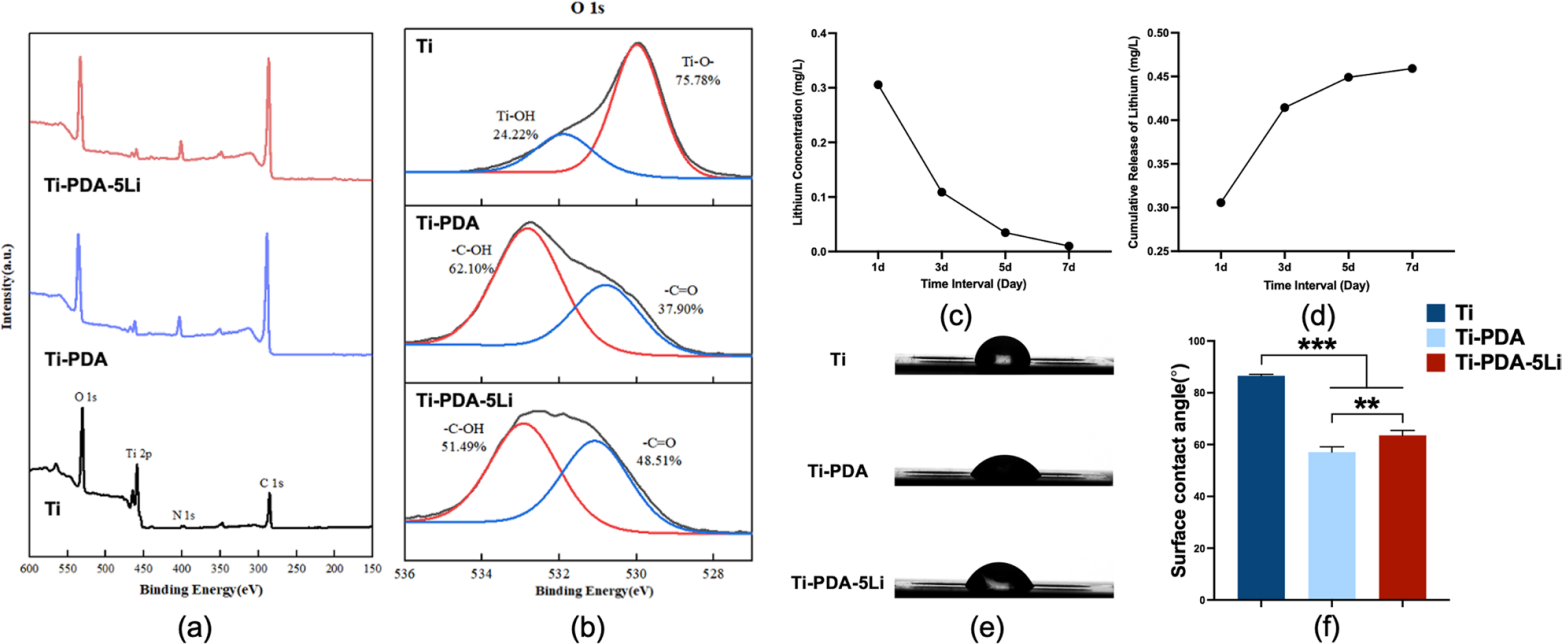
Surface characterization and Li^+^ release behavior of Li^+^ doped titanium alloy (Ti-PDA-5Li). **a** XPS survey spectra of Ti, Ti-PDA, and Ti-PDA-5Li groups, **b** High-resolution O 1s peak deconvolution for Ti, Ti-PDA, and Ti-PDA-5Li, **c** Daily Li^+^ release from Ti-PDA-5Li surface, **d** Cumulative Li^+^ release from Ti-PDA-5Li surface, **e** Water contact angle measurements and comparative analysis of surface hydrophilicity (**f**). Data: mean ± SD; **: *p* < 0.01, ***: *p* < 0.001 (one-way ANOVA)

XPS was used to analyze the surface chemistry of the samples. Full-spectrum scans and elemental composition data are shown in Fig. 5a; Table 3. The untreated titanium surface showed signals for Ti, O, C, and N. After PDA modification, the Ti-PDA group exhibited increased N 1s and C 1s peak intensities and a decreased Ti 2p signal, consistent with surface coverage by PDA. No Li^+^ signal was detected in the Ti-PDA-5Li group, primarily due to three key factors: firstly, the inherently low photoelectron emission efficiency of Li, attributed to its low atomic number, which places it near or below the detection limit of XPS; secondly, the potential sub-surface distribution of Li ions, which may be coordinated within the PDA matrix rather than at the immediate surface (within the top 5–10 nm probed by XPS), thereby reducing their effective surface concentration; and thirdly, the possible migration or mild volatility of Li under prolonged X-ray exposure in ultra-high vacuum, which could further attenuate an already weak signal.

**Table 3:** Elemental composition and ratios of the specimen surface

Substrate	C(At.om%)	O(Atom%)	Ti(Atom%)	*N*(Atom%)
Ti	39.88	41.44	15.75	2.93
Ti-PDA	68.73	22.32	1.56	7.39
Ti-PDA-5Li	68.34	23.47	1.51	6.68

High-resolution O 1s spectra (Fig. 5b) indicated that oxygen on the untreated surface primarily existed as Ti–OH and Ti–O bonds from TiO₂. The Ti-PDA group showed additional oxygen-containing functional groups, including phenolic hydroxyl (–C–OH) and quinone (–C = O) groups. In the Ti-PDA-5Li group, the –C–OH peak area decreased from 62.10% to 51.49%, while the –C = O peak area increased from 37.90% to 48.51% (Table 3), suggesting partial conversion of phenolic hydroxyl to quinone groups during Li^+^ chelation.

Li^+^ release profiling using ICP-MS showed an early release peak (0.306 mg/L) followed by a gradual decrease, with cumulative release reaching approximately 0.459 mg/L by day 7 (Fig. 5c, d). It is approximately 0.066 mmol/L LiCl, which is far lower than potential toxic doses (typically > 5 mmol/L) [26]. Hydrophilicity tests indicated that the untreated titanium alloy had the highest water contact angle (86.56 ± 0.47°), indicating hydrophobicity (Fig. 5e). Both Ti-PDA and Ti-PDA-5Li groups showed improved hydrophilicity, with Ti-PDA exhibiting better wettability than Ti-PDA-5Li (Fig. 5f), suggesting that Li^+^ incorporation partially counteracts the hydrophilic effect of PDA.

### Li^+^ loaded titanium alloy modulated inflammatory response and macrophage polarization

**Fig. 6:**
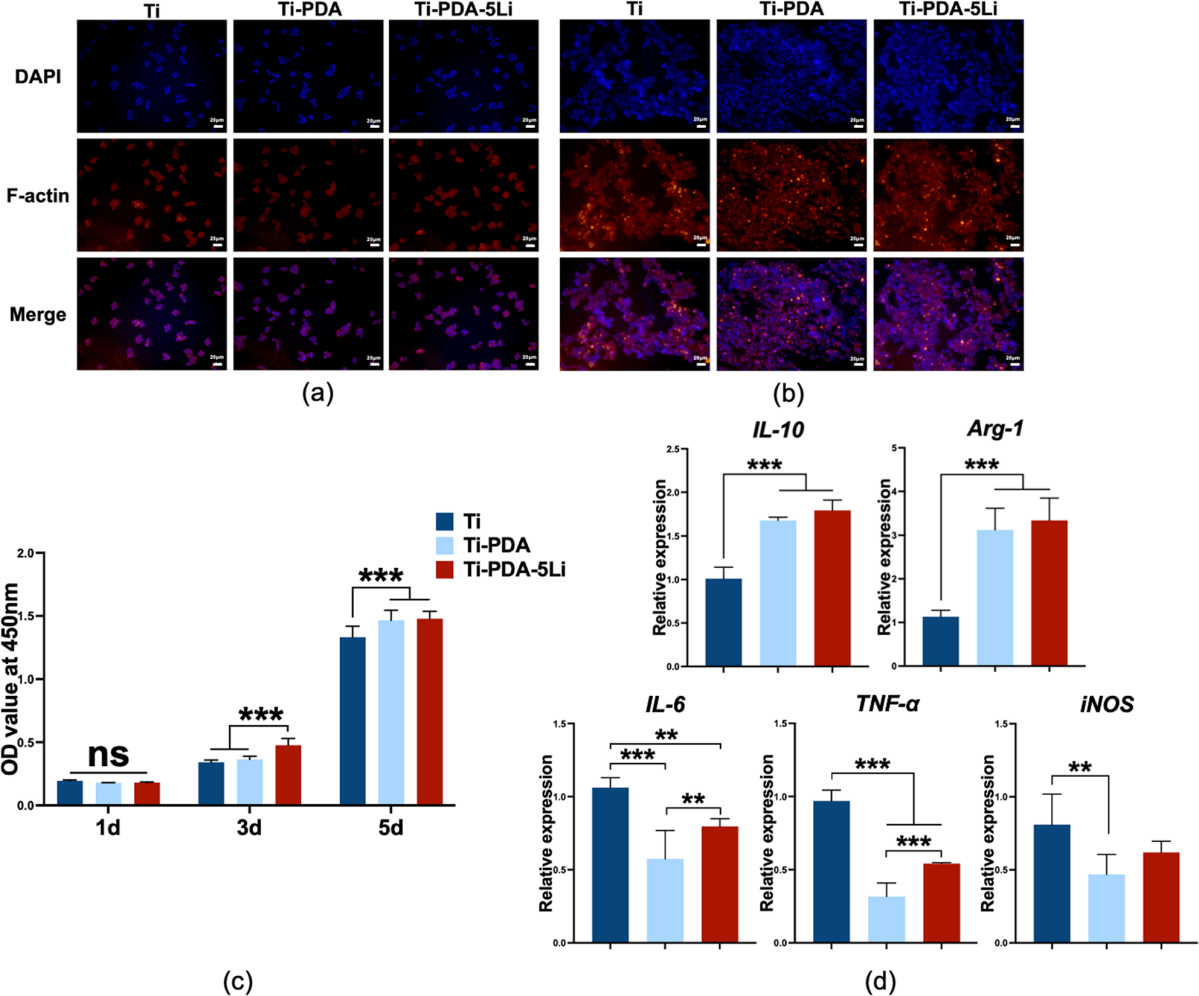
Effects of Ti-PDA-5Li on macrophage RAW264.7 adhesion, proliferation, and polarization. **a** Cytoskeleton staining of RAW264.7 cells cultured for 1 day (scale bar: 20 μm), **b** Cytoskeleton staining after 3-day culture (scale bar: 20 μm), **c** Proliferation rates of RAW264.7 cells, **d** Relative mRNA expression of inflammation and polarization-related genes (*IL-6*, *IL-10*, *TNF-α*, *iNOS*, *Arg-1*). Data: mean ± SD; **: *p* < 0.01, ***: *p* < 0.001 (one-way ANOVA with Tukey’s post-hoc test)

Phalloidin staining confirmed that RAW264.7 macrophages adhered normally to all surfaces (Ti, Ti-PDA, and Ti-PDA-5Li) with no significant morphological differences (Fig. 6a). Cells appeared small and round initially but proliferated and formed clusters over time (Fig. 6b). CCK-8 assays showed that macrophage proliferation increased on all materials, with Ti-PDA-5Li supporting significantly higher growth than other groups on day 3. By day 5, both modified surfaces outperformed pure Ti (Fig. 6c).

At the transcriptional level, Ti-PDA-5Li significantly downregulated pro-inflammatory cytokines (*IL-6*, *TNF-α*) and the M1 marker iNOS, while upregulating the anti-inflammatory cytokine *IL-10* and the M2 marker *Arg-1* (Fig. 6d). These results indicate that Ti-PDA-5Li supports macrophage adhesion and proliferation while promoting a shift from pro-inflammatory M1 to anti-inflammatory M2 polarization.

### Macrophages cultured on Li^+^ loaded titanium alloy enhanced osteogenic differentiation

**Fig. 7:**
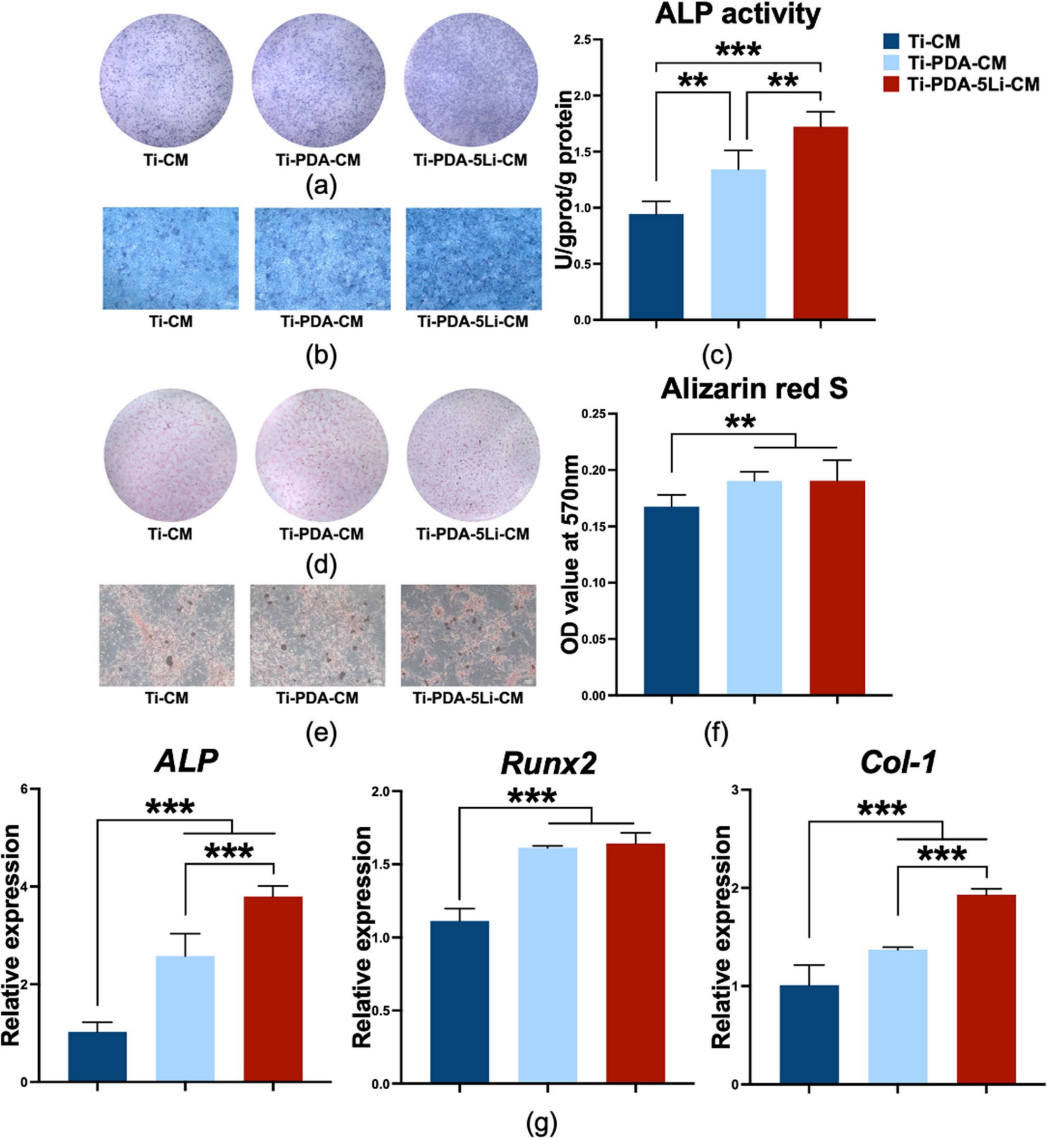
Effects of conditioned culture medium from macrophages co-cultured with Ti-PDA-5Li on osteogenic differentiation of rBMSCs. **a-c** ALP staining and activity assay of rBMSCs cultured with CM, **d-f** ARS staining and quantitative analysis of rBMSCs cultured with CM, **g** Detection of osteogenic differentiation-related gene transcription levels (*ALP*, *Runx2*, *Col-1*) in rBMSCs under CM treatment. Data: mean ± SD; **: *p* < 0.01, ***: *p* < 0.001 (one-way ANOVA with Tukey’s post-hoc test)

CM was collected from RAW264.7 macrophages cultured on different surfaces and used to treat rBMSCs. The Ti-PDA-5Li-CM group showed deeper ALP staining and higher ALP activity than other groups (Fig. 7a–c). After 14 days, both Ti-PDA-CM and Ti-PDA-5Li-CM groups promoted greater mineralized nodule formation compared to Ti-CM (Fig. 7d–f). qRT-PCR analysis revealed that osteogenic gene expression (*ALP*, *Runx2*, *Col-1*) was significantly upregulated in rBMSCs treated with CM from modified surfaces, with Ti-PDA-5Li-CM yielding the highest mRNA levels of *ALP* and *Col-1* (Fig. 7g).

## Discussion

Titanium meshes are widely utilized in alveolar bone regeneration for periodontitis-induced defects owing to their superior mechanical properties [4]. However, their inherent biological inertness limits their ability to directly regulate bone repair processes. Surface modification has emerged as an effective strategy to enhance the bioactivity of titanium implants [5]. In the present study, we employed PDA as a carrier to immobilize Li^+^ onto titanium alloy surfaces. Our results demonstrated that the Li^+^ loaded coating enabled sustained Li ions release, improved surface hydrophilicity, and significantly promoted osteogenic differentiation and immunomodulation, highlighting its potential for synergistic bone regeneration through direct osteogenic stimulation and immune microenvironment remodeling.

Surface modification of titanium alloys is essential for optimizing their biological performance. Current modification strategies can be broadly categorized into physical, chemical, and biochemical methods. Among these, biochemical approaches offer distinct advantages, including minimal dependence on substrate morphology or coating angle, low processing temperatures, reduced solvent usage, and the ability to incorporate thermo-sensitive biomaterials without compromising bioactivity [27]. Biochemical modifications can significantly enhance biocompatibility, improve tissue integration, confer antibacterial properties, and precisely regulate cellular behaviors [28].

In recent years, PDA has gained increasing attention as a versatile carrier for loading bioactive molecules or ions onto titanium surfaces [13, 14, 29]. PDA exhibits remarkable adhesion and adaptability, enabling uniform coating on various material geometries through a simple, eco-friendly process [30]. Its adhesion primarily relies on non-covalent interactions—supplemented by covalent bonding—with TiO_2_, with efficiency influenced by pH, humidity, and surface pretreatment [31]. Alkali-thermal treatment is a commonly used pretreatment method that generates vertically aligned sodium titanate nanocrystals on titanium substrates. This nanostructured surface not only increases specific surface area and adhesion strength but also facilitates subsequent biochemical functionalization [32]. While alkali-thermal treatment is well-established, studies combining PDA with active ion loading remain limited. In this work, we observed for the first time that after alkali-thermal treatment and LiCl incorporation via PDA, the titanium surface developed an open nano-needle-like porous architecture. Notably, pore-wall thickness increased with LiCl concentration, resulting in narrower nanopores. These porous structures provided favorable sites for LiCl loading and deposition.

The porous architecture generated by alkali-thermal treatment mimics the natural bone extracellular matrix (ECM), which provides mechanical support, mediates cell–matrix interactions, and regulates cellular functions [33]. Studies have shown that nanoscale topographic features on implant surfaces can effectively emulate native ECM structures, thereby influencing cell adhesion and differentiation [34]. In our study, both rBMSCs and RAW264.7 macrophages exhibited favorable early-stage adhesion on the modified surfaces, which may be partly attributed to this ECM-mimetic topography.

Enhanced cell adhesion is also closely associated with improved surface hydrophilicity following modification. Hydrophilic surfaces are known to promote protein adsorption in favorable conformations, thereby facilitating cell attachment [35]. The increased hydrophilicity of PDA-coated titanium alloys can be attributed to two factors: alkali-thermal treatment removes hydrophobic contaminants from the titanium surface [36], and PDA enhances the reactivity of the oxide layer [37]. Although the incorporation of Li^+^ in the Ti-PDA-5Li group slightly reduced hydrophilicity compared to Ti-PDA—possibly due to hindered water molecule interaction—it still outperformed unmodified titanium. This observation aligns with the maintained excellent cell adhesion on Ti-PDA-5Li, reinforcing the link between surface wettability and cellular behavior. Notably, despite its lower hydrophilicity relative to Ti-PDA, Ti-PDA-5Li demonstrated enhanced biological performance across multiple assays. This suggests that the positive effect of Li^+^ release on rBMSC activity may outweigh the influence of surface hydrophilicity, highlighting the predominant role of ionic modulation in driving the observed osteogenic response.

The osteogenic potential of Li^+^ has been increasingly documented [38, 39]. When immobilized on material surfaces, Li^+^ can be released gradually to suppress osteoclast activity, mitigate cartilage degeneration, and stimulate bone formation [40]. Previous studies suggest that Li^+^ promotes osteogenesis primarily via the Wnt/β-catenin signaling pathway [38], which activates downstream targets such as *Runx2*, a master transcription factor governing osteoblast differentiation. *Runx2* in turn upregulates key osteogenic markers including *ALP*, *OCN*, *OPN*, and *BSP*, driving matrix mineralization and bone remodeling [41]. Our findings are consistent with these mechanisms: the PDA-based Li^+^ coating enhanced rBMSC proliferation and upregulated mRNA expression of *Runx2*, *OCN*, *BSP*, and *OPN*, confirming the pro-osteogenic role of Li-modified titanium at the molecular level.

Implanted biomaterials often provoke immune responses in bone tissue [42]. Macrophages and their secreted cytokines serve as critical indicators of inflammatory reactions, with polarization between the pro-inflammatory M1 phenotype and the anti-inflammatory M2 phenotype reflecting immune status transitions [43]. M1 macrophages typically express high levels of *IL-1β*, *IL-6*, and *TNF-α*, initiating inflammatory cascades, whereas M2 macrophages upregulate *IL-10* and *TGF-β* to resolve inflammation [44]. In this study, the incorporation of Li^+^ into the PDA-modified titanium coating (Ti-PDA-5Li) elicited a distinct immunomodulatory response in RAW264.7 macrophages. Specifically, the Ti-PDA-5Li group significantly downregulated the expression of classic pro-inflammatory cytokines *IL-6* and *TNF-α*, while concurrently enhancing the secretion of the anti-inflammatory cytokine *IL-10*. At the phenotypic level, the coating suppressed the expression of *iNOS*, and promoted the expression of *Arg-1*. This coordinated shift in both secretory profile and marker expression strongly indicates that Ti-PDA-5Li promotes macrophage polarization towards the anti-inflammatory M2 state, thereby fostering an immunomodulatory microenvironment.

Macrophage plasticity is a central regulator of the healing cascade; the initial pro-inflammatory M1 phase is necessary for clearing debris and initiating repair, but a prolonged M1 response can be detrimental to osteogenesis by creating a hostile inflammatory milieu. Conversely, a timely transition to the M2 phenotype is crucial for resolving inflammation, promoting angiogenesis, and facilitating osteogenic differentiation of mesenchymal stem cells [45]. Our findings—that Ti-PDA-5Li attenuates M1-associated signals and amplifies M2-associated signals—suggest that the Li^+^ releasing coating actively guides this critical phenotypic switch. This immunomodulatory action likely establishes a favorable osteoimmune environment, which we posit is a key mechanism underlying the enhanced osteogenic outcomes observed in subsequent cellular experiments. This interpretation also aligns with established literature on the anti-inflammatory properties of Li^+^ [46, 47].

To further investigate how Li^+^ modified titanium influences bone regeneration via immune regulation, we used a CM-based indirect co-culture system [48] to simulate the in vivo immune microenvironment. Results showed that medium from macrophages cultured on Li^+^ loaded titanium (Ti-PDA-5Li-CM) enhanced the expression of osteogenic genes (*Runx2*, *ALP*, *Col-1*), ALP activity, and mineralization in rBMSCs. This suggests that the osteogenic improvement may stem from Li-induced M2 polarization and subsequent *IL-10* upregulation, which can activate osteogenic pathways such as MAPK/ERK and BMP/Smad [49, 50]. *IL-10* likely plays a central role in Li^+^ mediated immunomodulation for bone regeneration, though other pathways may also contribute and warrant further investigation.

Although this study demonstrates a significant association between Li-induced immunomodulation and enhanced osteogenesis, it must be emphasized that the mechanistic evidence remains correlative rather than definitively causal. The observed synergistic effect likely involves complex signaling cascades linking macrophage polarization to osteogenic differentiation. While our findings suggest that Li^+^ modified surfaces create an osteoimmunomodulatory microenvironment conducive to bone regeneration, several key mechanistic questions remain unresolved. First, the specific cytokine profile mediating these effects requires systematic characterization through proteomic analysis of conditioned media. Second, the intracellular signaling pathways activated in rBMSCs within this immunomodulated microenvironment need direct validation via pathway inhibition experiments. Future investigations should utilize neutralizing antibodies against candidate cytokines (e.g., anti‑IL‑10) and specific pathway inhibitors (e.g., ERK or Smad inhibitors) to establish causal relationships. Additionally, more sophisticated co‑culture models that permit direct cell‑cell contact could uncover further paracrine signaling mechanisms.

Finally, bone remodeling is essentially a dynamic coupling of osteogenic and osteoclastic activities. Although existing studies suggest that Li^+^ can inhibit osteoclast function [51], the specific effect of PDA-loaded Li^+^ on osteoclasts has not been investigated in this work. Furthermore, the stability of the coating under mechanical stress after lithium loading, the release kinetics of lithium ions (including release duration and concentration profile), and their biological effects in vivo are all critical for the eventual application of this material. Future studies will further explore the bidirectional regulatory effect of PDA‑Li^+^‑loaded titanium surfaces on osteogenesis and osteoclastogenesis, and systematically evaluate the mechanical stability of the PDA‑Li^+^ coating in physiological stress environments as well as its long‑term Li^+^ release behavior. Additionally, in vivo animal experiments will be necessary to validate the osteogenic efficacy and long‑term biocompatibility of this functionalized coating, so as to facilitate its translation toward clinical applications.

## Conclusions

This study confirms the feasibility and osteogenic efficacy of a PDA-assisted Li⁺ loading strategy on titanium alloy surfaces under in vitro conditions. The findings not only offer a promising approach for optimizing the surface architecture of titanium implants but also underscore the potential of concurrently modulating immune response and osteogenic activity to promote bone regeneration around implants. Compared with alternative surface modification techniques, this PDA-mediated strategy achieves efficient Li⁺ loading in an aqueous environment under mild conditions—requiring neither complex instrumentation nor harsh reaction parameters. The procedure is simple, utilizes minimal intermediate reagents, and avoids the need for multiple organic solvents, thereby enhancing both operational practicality and environmental compatibility. Collectively, this work provides a feasible and efficient pathway for designing titanium alloy implant surfaces that integrate enhanced bioactivity with clinical translatability.

## Data Availability

The datasets used and/or analyzed during the current study are available from the corresponding author on reasonable request.
